# The Economic Burden of Brucellosis Care in China: Socioeconomic Status Inequality

**DOI:** 10.1155/2024/7992287

**Published:** 2024-08-03

**Authors:** Hai-Bo He, Qiao-Shan Lu, Lian-Lian Wang, Muhetal Aishan, Jiang-Shan Zhao, Xian-Yan Tang, Man-Tong Zhu, Milikam Reheman, Qiu-Lan Chen, Yan-Ping Zhang

**Affiliations:** ^1^ Prevention and Control Institute of Parasitic Diseases and Brucellosis Xinjiang Uygur Autonomous Region Centers for Disease Control and Prevention, Urumqi, China; ^2^ School of Public Health Guangxi Medical University, Nanning, China; ^3^ School of Nursing Peking University, Beijing, China; ^4^ Wushi County Center for Disease Control and Prevention, Aksu, China; ^5^ National Key Laboratory of Intelligent Tracking and Forecasting for Infectious Diseases Chinese Center for Disease Control and Prevention, Beijing, China

## Abstract

The economic burden of brucellosis care on patients can lead to significant financial strain, despite partial coverage by medical insurance. However, there is limited research on the out-of-pocket costs faced by brucellosis patients. Therefore, our study aimed to investigate the costs and out-of-pocket expenses of brucellosis care, specifically examining the varying socioeconomic status of patients in Xinjiang, China. We collected cost and demographic data from 563 respondents and their hospital bills and employed latent variable analysis to assess socioeconomic status. The majority of patients belonged to the middle and lower socioeconomic status categories (85.97%), and they were primarily farmers and herders (82.77%). The median direct cost per brucellosis episode was USD 688.65, with out-of-pocket expenses amounting to USD 391.44. These costs exceeded both the 2020 Xinjiang and national per capita health expenditures (USD 233.66 and USD 267.21, respectively). Notably, the overall medical reimbursement rate was 48.60%, and for outpatient costs, it was merely 12.82%. Despite higher out-of-pocket costs among high socioeconomic status patients, the percentage of income spent was higher (37.23%) for patients in the lower socioeconomic status group compared to other groups (16.25% and 12.96%). In conclusion, our findings highlight that brucellosis patients are predominantly from the middle and lower socioeconomic status, with high out-of-pocket expenses placing them under significant financial pressure. Moreover, there is notable inequity in economic consequences across different socioeconomic status groups. These results call for policy interventions aimed at reducing brucellosis-related poverty and promoting equitable access to care.

## 1. Background

Brucellosis, caused by bacteria of the genus Brucella, is a highly neglected zoonotic disease. It is reported to affect over 500,000 human cases annually in more than 170 countries and regions worldwide [1–3]. In the Xinjiang Uygur Autonomous Region (Xinjiang), China, brucellosis poses a significant public health concern, with an incidence rate of 18.50 per 10,000 in 2021, ranking among the top three regions in China [4]. Although the mortality rate of brucellosis is low, the disease can impact multiple organ systems and have adverse effects on household productivity, leading to a significant economic burden for individual patients and the healthcare system [3, 5, 6]. Given Xinjiang's role as a pivotal center for the livestock industry and a significant province in China's poverty alleviation initiatives, it is imperative to conduct an assessment of the economic impact of human brucellosis in order to sustain the progress made in poverty reduction and safeguard the livelihoods of the population.

The availability of medical insurance coverage for brucellosis diagnosis and treatment in China is limited, with varying reimbursement rates among different hospital levels [7]. This, combined with the atypical presentation of brucellosis, often leads to diagnostic delays. Lower-level hospitals face challenges in diagnosing and treating the disease, resulting in the transfer of critically ill patients to higher-level facilities [8]. As a consequence, patients incur significant out-of-pocket (OOP) expenses and additional costs for travel and subsistence, particularly in underdeveloped areas of Xinjiang. Furthermore, brucellosis was not included in the list of chronic diseases at the time of the study. As a result, the outpatient costs exceeded the coverage provided by basic medical insurance in Xinjiang, forcing patients to fully bear the remaining expenses below the payment threshold. This adds to the financial burden on individual patients. Therefore, it is important to evaluate the OOP payments of brucellosis patients and assess the economic burden among hospitals and different economic regions in Xinjiang. This knowledge is crucial for optimizing the medical reimbursement policy specific to brucellosis patients in Xinjiang, preventing them from falling into poverty as a result of the disease, and ensuring that Xinjiang maintains its progress in poverty eradication efforts.

The impact of socioeconomic factors on health and well-being is substantial, leading to greater disparities and inequalities in healthcare. Lower socioeconomic status (SES), influenced by poor living conditions and financial limitations, among other factors, heightens the susceptibility to brucellosis infection, comorbidities, and unfavorable treatment outcomes. Individuals with lower SES often confront economic difficulties that can worsen and perpetuate a cycle of poverty [9, 10].

Previous studies on brucellosis have primarily examined the overall economic losses [11, 12] and the medical costs [13–16] associated with the disease. However, these studies have not specifically addressed the OOP costs incurred by individual patients, particularly within different SES. As a result, the true economic burden of brucellosis on patients may have been unclear and overlooked. In light of this, the objective of this study is to investigate the costs associated with the care of brucellosis, assess the economic burden across different economic regions and levels of hospitals, and evaluate the financial implications for individuals with varying SES.

## 2. Methods

### 2.1. Study Design and Setting

**Figure 1:**
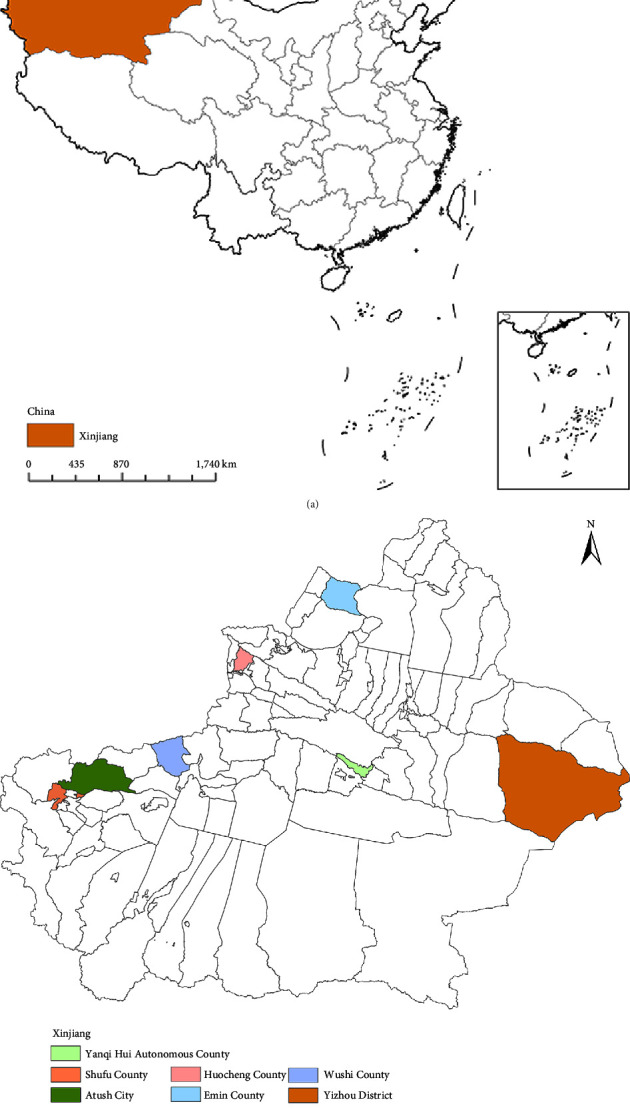
Geographic location. (a) Xinjiang, China. (b) Study settings: Huocheng, Atushi, Emin, Yanqi, Yizhou, Wushi, and Shufu.

A cross-sectional study was conducted in seven counties in Xinjiang, China, namely, Huocheng, Atushi, Emin, Yanqi, Yizhou, Wushi, and Shufu from April to June 2021 (Figure 1). These counties were randomly selected in the Chinese National Notifiable Disease Reporting System (NNDRS) according to their incidence of brucellosis. The study followed the STrengthening the Reporting of OBservational studies in Epidemiology (STROBE) for cross-sectional studies guidelines (Supplementary File 2). The study protocol was approved by the Research Ethics Review Committee of the Chinese Center for Disease Control and Prevention (Approval number: 202120), and signed informed consent was obtained from all participants prior to the investigation. For individuals under the age of 18, we obtain informed consent from their legal guardians.

#### 2.1.1. Sample Size

The “Confidence Intervals for One Proportion” module from Power Analysis and Sample Size Software (Version 15, NCSS LLC., East Kaysville, Utah, United States) was used to perform the sample size calculation. Based on the assumption of a 20% prevalence of patients with OOP exceeding their per capita annual household income, there were a precision of 0.07, an *α* level of 0.05, and a 5% dropout rate. The calculated sample size was 557.

### 2.2. Sampling and Participants

A multistage random sampling method was utilized to select aged 15 years or older who had been diagnosed with brucellosis. These individuals had completed full treatment for a single episode and were reported in the NNDRS between January 1, 2019, and March 1, 2021, in order to fulfill the sample size criteria. The diagnosis of brucellosis was based on the criteria outlined in the diagnosis of brucellosis (WS 269-2019) [17].

**Figure 2:**
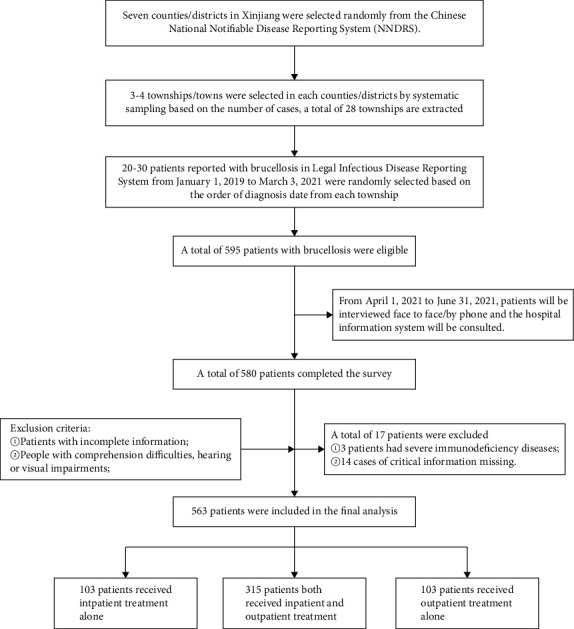
Brucellosis patient screening process.

The sampling process was performed in three steps: in step 1, seven counties/districts in Xinjiang were randomly chosen from the NNDRS. In step 2, each township within these counties/districts was assigned a unique number based on the number of cases. Systematic sampling was then used to select 3-4 townships in each county/district. In step 3, from each selected township, 20–30 patients were randomly chosen based on the order of their diagnosis date (Figure 2).

This study was community-based, conducted by trained interviewers, using a uniform questionnaire to interview the subjects face by face in the communities where the selected brucellosis patients lived. The uniform questionnaire was used to gather the following information: (1) demographic and socioeconomic characteristics of the subjects such as age, sex, education, occupation, and per capita annual household income; (2) direct medical cost such as inpatient costs, outpatient costs, medical expenses covered by insurance system, and out-of-pocket costs (OOPO) associated with each brucellosis episode of care; (3) nonmedical expenses associated with transportation and accommodation. As Xinjiang has designated hospitals for brucellosis patients for appropriate treatment, normally in county or township level hospitals, and the electronic degree of these grass-roots hospitals is very high, these direct medical costs can be obtained from the Hospital Electronic Information System. Patients can be reimbursed at hospital and only need to pay the OOPP for the medical expense.

A standardized protocol was implemented to ensure consistency in interviewer training and quality control supervision throughout all survey instances. The interviewers included 2 provincial CDC staff and 12 trained staff from the local Centers for Disease Control and Prevention. In addition, 3–5 local volunteers who were familiar with the customs and languages of the patients provided assistance in administering the questionnaires. Each questionnaire underwent a thorough review by qualified supervisory staff. Data management specialists checked the collected questionnaires for completeness and logical consistency.

### 2.3. Cost Measurement

Direct costs can be classified into medical costs and nonmedical costs. Medical costs primarily consist of expenses related to self-purchased medications, outpatient diagnostic and treatment services, and hospitalization during patient treatment. On the other hand, nonmedical costs encompass additional expenses such as transportation, lodging, and food. Out-of-pocket medical costs refer to the portion of medical costs that patients must pay directly after reimbursement within a single episode of care. Out-of-pocket costs can be calculated as the sum of out-of-pocket medical costs and nonmedical costs.

### 2.4. Measurement of Socioeconomic Status and Regional Classification

SES is a key determinant of health, as it encompasses access to material, human, and social resources. Education, employment, and income are vital components of SES and can be combined to derive an SES index [18]. In this research, the SES of patients was assessed using latent class analysis (LCA) based on their income, education, and occupation [19, 20]. Please refer to Supplementary Appendix 1 (Supplementary File 1) for further details.

Seven counties/districts have been categorized into low-, middle-, and high-economic regions based on their regional GDP and the per capita disposable income of both rural and urban residents in 2020 [21].

### 2.5. Data Analyses

We employed the statistical software package RX64 4.3.0 to undertake data analysis. Descriptive statistics and percentage distributions were utilized, with median and interquartile ranges (IQR) presented. All monetary values were estimated in United States dollars (USD) using a currency exchange rate of Chinese Yuan (CNY) 689.76 to USD 100 in 2020 [22]. Patients were categorized into four quartiles (Q1 to Q4) based on their per capita annual household income. Categorical data were summarized as proportions, and the *x*^2^ test was employed to assess differences. The Kruskal–Wallis test was used to examine variations in various costs and proportions.

## 3. Results

In total, 595 patients met the criteria for participation in the study, and of these, 580 patients successfully completed the survey. After excluding 17 patients from the analysis, a total of 563 patients were included in the final analysis (Figure 2).

### 3.1. Sociodemographic Characteristics

The majority of brucellosis cases in this study were male (70.52%), of working age (76.20% aged 25–59), mainly belonging to the Uyghur ethnic group (51.69%), engaged in farming (59.15%), and had less than a high school education (89.34%). Approximately 45.47% of patients had below-median per capita annual household income, and 48.67% resided in southern Xinjiang. Among the patients, 55.95% received both outpatient and inpatient care, with 30.55% being treated in tertiary hospitals. Overall, 86.86% of patients were in the acute stage, 40.85% experienced complications, and 12.08% had other underlying diseases. Notably, 85.97% of patients were classified as having low-to-middle SES, and these low-to-middle SES patients were mostly farmers and herders and had lower education levels compared to patients with high SES. Patients from different SES groups showed significant disparities in terms of geographic location, hospitals utilized, and treatment methods (all *p* values <0.05), while no significant differences were observed in age, gender, ethnicity, and clinical information (Table 1).

### 3.2. Costs of Brucellosis Care

**Figure 3:**
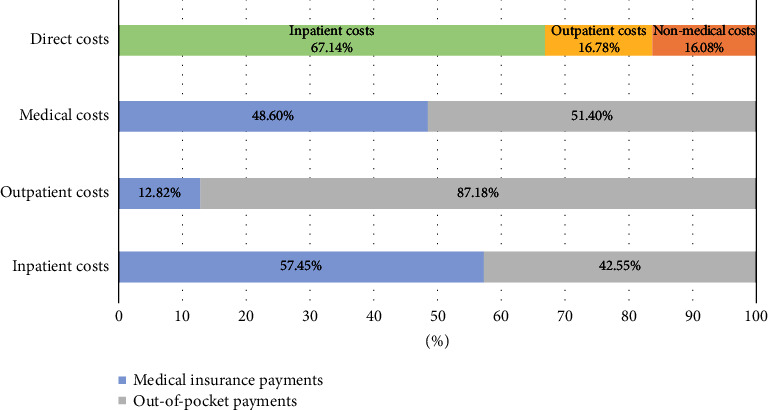
Brucellosis-related medical costs and payment components.

The median direct costs for the entire brucellosis episode were USD 688.65, with an interquartile range (IQR) of 333.45 to 1563.54. Medical costs were significantly higher than nonmedical costs, with median values of USD 541.28 and USD 101.48, respectively. The median out-of-pocket (OOP) costs amounted to USD 391.44, with an IQR of 202.97 to 939.46. These figures were considerably higher than the per capita healthcare expenditure in Xinjiang and China in 2020, which were USD 233.66 and USD 267.21, respectively. It is worth noting that the overall reimbursement rate for medical costs was only 48.60%, and health insurance only reimbursed 12.82% of outpatient costs (Table 2 and Figure 3).

### 3.3. Costs of Brucellosis across SES

**Figure 4:**
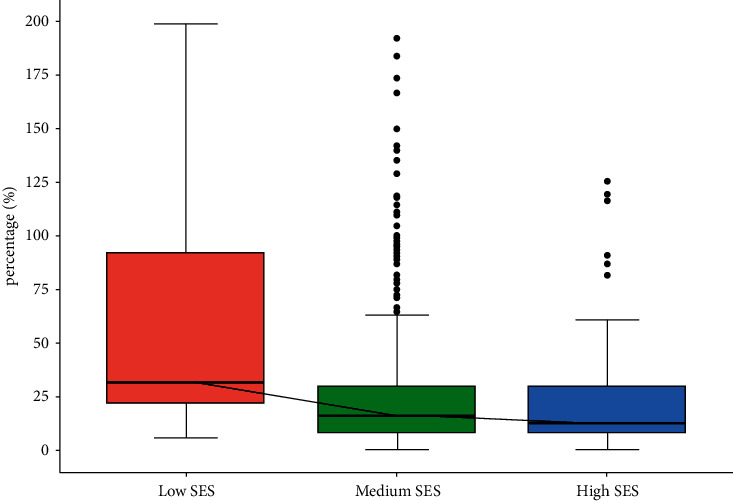
OOP (out-of-pocket) costs as a percentage of reported per capita annual household income across socioeconomic statuses (SESs).

The OOP costs accounted for 19.88% (IQR: 9.55%, 40.00%) of the per capita annual household income. Interestingly, while patients with higher SES experienced the highest OOP costs, patients with lower SES reported a higher percentage of their income being spent on healthcare expenses (37.23% [IQR: 22.73%, 131.58%]) compared to other SES groups (16.25% and 12.96%). This discrepancy highlights the significant financial burden faced by individuals from lower socioeconomic backgrounds, even with comparatively lower OOP costs (Table 2 and Figure 4).

### 3.4. Costs of Brucellosis in Different Regions and Hospitals

In regions with low-economic status, the average annual household income per person was found to be the lowest at USD 1532.42. Interestingly, these regions also faced the highest direct costs (USD 1764.97) and OOP expenses (USD 1075.74). The burden on tertiary hospitals and their patients was particularly high, particularly with respect to medical costs (USD 1377.68) and OOP expenses (USD 1126.48), surpassing those in lower-level hospitals. The proportion of OOP expenses for inpatient costs increased as the level of hospitals increased, but there was minimal difference in outpatient costs (Tables 3 and 4).

## 4. Discussion

Brucellosis has been demonstrated to have significant negative socioeconomic impacts, with the majority of losses attributed to livestock [11]. Studies have shown that brucellosis impacts society economically by affecting livestock's reproductive rates, milk production, and overall health status [23]. However, within the framework of “One Health,” the cost assessment of brucellosis should also consider the costs associated with human diseases. Therefore, further evaluation of the overall and individual economic burdens incurred during the treatment of human brucellosis is necessary.

This study aimed to examine the economic impact of brucellosis on patients in Xinjiang. The findings revealed a substantial financial burden, with brucellosis patients incurring median OOP costs that exceeded both the per capita health expenditures in Xinjiang and on a national level in 2020 [24]. Moreover, this economic burden was further exacerbated by socioeconomic disparities, with economically disadvantaged patients experiencing more severe financial strain. Tertiary hospitals and those located in low-economic regions were particularly affected by higher financial burdens. Notably, while the proportions of OOP costs for inpatient care varied across hospitals, no significant differences were observed for outpatient care.

### 4.1. Interpretation of Key Findings

#### 4.1.1. Costs of Brucellosis

The study found that the median direct costs for managing brucellosis were USD 688.65 (IQR: 333.45, 1563.54), indicating a significant financial impact associated with this infectious disease. The median medical costs for brucellosis (USD 541.58) were higher than the nonmedical costs (USD 101.48). This finding is consistent with a previous study conducted in Jingyuan, Gansu, where the median medical costs were USD 1715.81 compared to USD 268.21 for nonmedical costs [16]. Additionally, the median OOP costs for brucellosis were USD 391.44 (IQR: 202.97, 939.46), which were considerably higher than the per capita healthcare expenditure in Xinjiang (USD 233.66) and China (USD 267.21) in 2020 [24]. This indicates that residents in Xinjiang face a significant financial strain in the treatment of brucellosis. When considering previous cost analysis research on animal brucellosis, the economic impact of this disease may pose a substantial damage to the region's agricultural economy.

The total rate of medical reimbursement in this study was 48.60%, significantly lower than the coordinated fund for basic medical insurance for urban and rural residents in Xinjiang at the end of 2020 (68.10%) [25]. This discrepancy may be attributed to the lack of coverage for all outpatient treatments for human brucellosis under medical insurance in Xinjiang during the study period, resulting in a very low outpatient reimbursement rate (12.82%). This highlights a potential inadequacy in Xinjiang's brucellosis medical insurance policy, which could impose a heavy financial burden on patients who require frequent outpatient visits, particularly those with chronic conditions. Fortunately, in November 2023, the Xinjiang government added brucellosis to the list of outpatient chronic diseases covered by the basic medical insurance for the Xinjiang Uygur Autonomous Region [26]. Consequently, outpatient brucellosis services will no longer have a deductible, and the reimbursement limit will be significantly increased. This important development is expected to substantially alleviate the financial burden on patients.

### 4.2. SES Inequality in Economic Consequences of Brucellosis

The results of our study indicate that individuals affected by brucellosis in Xinjiang are primarily from low and middle SES backgrounds. Their median per capita annual household income was found to be below the national lower middle-income group (USD 2383.87), at USD 956.85 and USD 1742.64, respectively [27]. The majority of these individuals work as farmers or herders, and they have lower levels of education and social status. They also have limited sources of income, which contributes to their economic disadvantages in effectively responding to the challenges posed by brucellosis.

Brucellosis is a disease associated with poverty, and it particularly affects the most economically disadvantaged individuals [28]. In our study, we found that patients with higher SES had higher OOP costs compared to those with lower SES. However, when considering OOP costs as a percentage of per capita annual household income, the burden decreased with higher SES. These findings are consistent with a previous study on tuberculosis patients, which showed that patients with low ability to pay and that even low-cost medical care could significantly impact their families [7]. Therefore, socioeconomic disparities worsen the economic consequences of brucellosis, placing a greater financial burden on economically disadvantaged patients.

### 4.3. Brucellosis Economic Burden: Disparities in Regions and Hospitals

The healthcare burden is highest in tertiary hospitals and regions with low-economic levels, which may be attributed to the unequal distribution of healthcare resources. Regions with low income, particularly those in remote areas, often face limited access to medical resources [29]. This lack of resources leads to higher expenses for transportation and accommodation when seeking care, resulting in discouragement and delays in seeking care. Tertiary hospitals, being equipped with better medical resources, usually handle high-risk patients requiring urgent and comprehensive care, resulting in increased medical costs. Differences in charging policies and reimbursement limits among hospitals contribute to variations in the OOP proportions for healthcare expenses [30, 31]. Our study shows that primary hospitals have the lowest OOP proportions for inpatient costs, while tertiary hospitals have the highest proportions, which is consistent with current health policy trends [30]. However, despite efforts to align with comprehensive health care provision, reimbursement for outpatient services remains limited even in primary and lower-level Medicare Priority Hospitals. This indicates challenges in expanding comprehensive healthcare beyond inpatient care.

## 5. Policy Implications

Xinjiang was found to bear a significant economic burden in the context of brucellosis care. The majority of brucellosis patients in this region were from middle to lower socioeconomic status, and the high out-of-pocket expenses related to brucellosis care imposed substantial financial pressure on them. It is evident that the existing medical insurance policies fall short in terms of providing sufficient financial risk protection for these patients. Therefore, it is imperative to implement measures that aim at reducing and compensating for the medical costs borne by patients. This could involve lowering the deductible threshold for brucellosis treatment and expanding the coverage of outpatient services in the medical insurance program. In addition, policymakers should also address the equitable distribution of healthcare resources among regions and hospitals to ensure a fair sharing of the healthcare burden. The government needs to improve preventive measures and enhance the diagnostic and treatment capabilities of primary hospitals to diminish the hospitalization rate among brucellosis patients. Finally, when formulating welfare and protection policies for brucellosis patients, the socioeconomic inequality should be taken into account to effectively alleviate the financial burden on vulnerable groups.

### 5.1. Study Limitations

The study acknowledges certain limitations that warrant consideration. Owing to the absence of standardized criteria for precisely estimating such costs, the analysis did not encompass indirect expenses linked to brucellosis patients, such as productivity losses resulting from absenteeism. Consequently, there may have been an underestimation of the economic impact of brucellosis on patients. Future studies should employ internationally standardized cost measurement methods to comprehensively assess the financial burden of human brucellosis. As a result, the economic burden of brucellosis on patients may have been underestimated. Future studies should employ internationally standardized cost measurement methods to comprehensively assess the financial burden of human brucellosis. Additionally, due to the retrospective nature of our study, recall bias was unavoidable. To minimize its influence, we chose to concentrate on the most recent episode of brucellosis for each participant.

## 6. Conclusion

Brucellosis imposes a significant economic burden on both the healthcare system and affected individuals. Patients with low socioeconomic status are particularly vulnerable to the inequitable economic consequences of this disease. Targeted financial and social support for disadvantaged groups has been shown to effectively alleviate the economic burden experienced by brucellosis patients. Furthermore, it is recommended to optimize the health insurance structure by reducing the deductible threshold for brucellosis treatment and expanding the coverage of medical insurance services. Propoor programs should also be implemented to prevent brucellosis patients from falling into the medical poverty trap.

## Figures and Tables

**Table 1:** Sociodemographic and clinical characteristics of participants.

Variable	Total *n* = 563 (100%)	Socioeconomic Status (SES)			*x* ^2^	*p* value
		Low *n* = 143 (25.40%)	Medium *n* = 341 (60.57%)	High *n* = 79 (14.03%)		
Age groups in years (%)					7.497	0.112
≤24	25 (4.44)	6 (4.20)	13 (3.81)	6 (7.59)		
25–59	429 (76.20)	111 (77.62)	253 (74.19)	65 (82.28)		
≥60	109 (19.36)	26 (18.18)	75 (21.99)	8 (10.13)		
Gender (%)					0.986	0.611
Male	397 (70.52)	102 (71.33)	236 (69.21)	59 (74.68)		
Female	166 (29.48)	41 (28.67)	105 (30.79)	20 (25.32)		
Ethnicity (%)					11.190	0.191
Han	39 (6.93)	10 (6.99)	25 (7.33)	4 (5.06)		
Uyghur	291 (51.69)	80 (55.94)	164 (48.09)	47 (59.49)		
Kazakh	87 (15.45)	13 (9.09)	62 (18.18)	12 (15.19)		
Hui	64 (11.37)	21 (14.69)	37 (10.85)	6 (7.59)		
Others	82 (14.56)	19 (13.29)	53 (15.54)	10 (12.66)		
Occupation (%)					434.278	0.000
Jobless	11 (1.95)	3 (2.10)	8 (2.35)	0 (0.00)		
Farmer	333 (59.15)	94 (65.73)	237 (69.50)	2 (2.53)		
Herdsman	133 (23.62)	30 (20.98)	96 (28.15)	7 (8.86)		
Livestock-related workers	31 (5.51)	14 (9.79)	0 (0.00)	17 (21.52)		
Veterinarian	15 (2.66)	2 (1.40)	0 (0.00)	13 (16.46)		
Civil servants, teachers, doctors, etc.	40 (7.10)	0 (0.00)	0 (0.00)	40 (50.63)		
Education level (%)					248.584	0.000
Illiterate	40 (7.10)	14 (9.79)	25 (7.33)	1 (1.27)		
Primary school	265 (47.07)	90 (62.94)	169 (49.56)	6 (7.59)		
Junior high school	198 (35.17)	38 (26.57)	133 (39.00)	27 (34.18)		
High school/technical secondary school	38 (6.75)	1 (0.70)	14 (4.11)	23 (29.11)		
College degree or above	22 (3.91)	0 (0.00)	0 (0.00)	22 (27.85)		
Geographical location^a^ (%)					6.088	0.048
Northern Xinjiang	289 (51.33)	72 (50.35)	186 (54.55)	31 (39.24)		
Southern Xinjiang	274 (48.67)	71 (49.65)	155 (45.45)	48 (60.76)		
Income quartiles^b^ (%)					660.166	0.000
Q1	140 (24.87)	140 (97.90)	0 (0.00)	0 (0.00)		
Q2	116 (20.60)	1 (0.70)	114 (33.43)	1 (1.27)		
Q3	140 (24.87)	2 (1.40)	127 (37.24)	11 (13.92)		
Q4	167 (29.66)	0 (0.00)	100 (29.33)	67 (84.81)		
Complications^c^ (%)					5.280	0.071
No	333 (59.15)	73 (51.05)	210 (61.58)	50 (63.29)		
Yes	230 (40.85)	70 (48.95)	131 (38.42)	29 (36.71)		
Counderlying disease (%)	s				4.073	0.130
No	495 (87.92)	119 (83.22)	306 (89.74)	70 (88.61)		
Yes	68 (12.08)	24 (16.78)	35 (10.26)	9 (11.39)		
Clinical stage^d^ (%)					7.794	0.099
Acute stage (<3 months)	489 (86.86)	117 (81.82)	305 (89.44)	67 (84.81)		
Subacute stage (3–6 months)	33 (5.86)	13 (9.09)	13 (3.81)	7 (8.86)		
Chronic stage (>6 months)	41 (7.28)	13 (9.09)	23 (6.74)	5 (6.33)		
Treatment (%)					21.449	0.000
Inpatient	103 (18.29)	12 (8.39)	66 (19.35)	25 (31.65)		
Outpatient	145 (25.75)	35 (24.48)	90 (26.39)	20 (25.32)		
Inpatient and outpatient	315 (55.95)	96 (67.13)	185 (54.25)	34 (43.04)		
Hospital level (%)					23.463	0.001
Primary and below hospital	61 (10.83)	23 (16.08)	37 (10.85)	1 (1.27)		
Secondary hospital	188 (33.39)	33 (23.08)	120 (35.19)	35 (44.30)		
Tertiary hospital	172 (30.55)	55 (38.46)	92 (26.98)	25 (31.65)		
Unknown	142 (25.22)	32 (22.38)	92 (26.98)	18 (22.78)		

^a^Xinjiang was divided into South Xinjiang and North Xinjiang by the Tianshan Mountains. ^b^Income quartiles were arranged from lower to higher (Q1 = lower; Q4 = higher). ^c^Complications include those involving the musculoskeletal, respiratory, nervous, and other systems. ^d^The acute stage was defined as the period within three months from onset to diagnosis, the subacute stage spans three to six months, and the chronic stage extends beyond six months. Abbreviations: Q1: 1st quartiles, Q2: 2nd quartiles, Q3: 3rd quartiles, Q4: 4th quartiles.

**Table 2:** Brucellosis-related costs (USD)^a^ across the socioeconomic status (SES).

Indicators	Total *n* = 563 (100%)	Socioeconomic Status (SES)			*H*/*Z*	*p* value
		Low *n* = 143 (25.40%)	Medium *n* = 341 (60.57%)	High *n* = 79 (14.03%)		
Direct costs	688.65 (333.45, 1563.54)	565.41 (297.78, 1809.20)	650.65 (327.65, 1376.13)	985.85 (618.30, 2274.69)	23.622	0.000^*∗*^
Medical costs	541.28 (260.96, 1289.93)	482.78 (246.46, 1468.63)	521.92 (251.54, 1165.91)	838.39 (374.90, 1825.26)	19.865	0.000^*∗*^
Inpatient costs	686.97 (260.96, 1379.01)	714.31 (202.97, 1499.14)	608.91 (260.96, 1159.82)	860.65 (413.78, 1884.71)	8.872	0.012^*∗*^
Outpatient costs	144.98 (72.49, 269.22)	144.98 (72.49, 260.96)	139.18 (72.49, 267.34)	234.86 (139.18, 434.93)	17.456	0.000^*∗*^
Nonmedical costs	101.48 (43.49, 240.66)	72.49 (30.45, 260.96)	86.99 (38.42, 217.47)	202.97 (101.48, 333.45)	19.338	0.000^*∗*^
OOP medical costs	251.61 (125.77, 682.85)	347.95 (195.72, 1246.31)	342.15 (188.47, 762.93)	652.40 (356.77, 1063.64)	26.062	0.000^*∗*^
OOP inpatient costs	217.47 (68.81, 622.82)	217.47 (43.49, 929.29)	188.47 (65.24, 504.07)	312.76 (144.98, 710.03)	11.791	0.003^*∗*^
OOP outpatient costs	115.98 (55.85, 246.46)	144.98 (72.49, 260.96)	104.38 (50.74, 217.47)	184.84 (52.19, 412.10)	10.719	0.005^*∗*^
OOP costs	391.44 (202.97, 939.46)	261.14 (138.60, 925.08)	218.92 (115.98, 558.94)	420.44 (180.13, 869.87)	22.815	0.000^*∗*^
OOP/medical costs (%)	54.02 (33.33, 82.16)	60.00 (43.59, 89.43)	51.10 (32.12, 81.30)	45.06 (31.63, 71.84)	0.217	0.897
OOP/direct costs (%)	63.64 (45.80, 87.88)	68.40 (54.65, 90.09)	61.73 (44.11, 87.05)	61.80 (42.86, 88.82)	0.306	0.858
Per capita annual household income	1739.74 (1203.32, 2899.56)	956.85 (869.87, 1087.33)	1742.64 (1478.78, 2899.56)	5074.23 (3131.52, 5799.12)	98.260	0.000^*∗*^
OOP costs/income^b^ (%)	19.88 (9.55, 40.00)	37.23 (22.73, 131.58)	16.25 (8.33, 31.52)	12.96 (7.10, 30.00)	13.713	0.001^*∗*^
Per capita healthcare expenditure in Xinjiang in 2020^c^	233.66					
Per capita healthcare expenditure in China in 2020^c^	267.21					

^a^Currency exchange rate: CNY 689.76 to USD 100. ^b^The income indicator is measured by per capita annual household income. ^c^Data from the 2020 China Health Statistics Yearbook. Abbreviations: *OOP*: out-of-pocket; *USD*: United States Dollar; *SES*: socioeconomic Status. ^*∗*^*p* value <0.05.

**Table 3:** Brucellosis-related costs (USD) in regions and hospitals.

	Per capita annual household income	Direct costs	Medical costs	Nonmedical costs	OOP costs
Regions^a^					
Low-economic regions *n* = 161 (28.60%)	1764.97 (810.13, 2753.09)	1764.97 (810.13, 2753.09)	1400.62 (716.36, 2234.71)	217.47 (86.99, 434.93)	1075.74 (434.93, 1853.91)
Medium-economic regions *n* = 292 (51.87%)	453.56 (251.03, 835.43)	453.56 (251.03, 835.43)	399.41 (217.47, 724.89)	46.39 (29.00, 115.98)	251.97 (159.48, 485.19)
High-economic regions *n* = 110 (19.54%)	809.97 (421.75, 1450.72)	809.97 (421.75, 1450.72)	507.94 (229.99, 1013.01)	217.47 (144.98, 335.26)	455.18 (281.11, 898.29)
*Z*	−11.759	−11.759	−12.415	−5.417	−8.703
*p-*value	0.000^*∗*^	0.000^*∗*^	0.000^*∗*^	0.000^*∗*^	0.000^*∗*^
Hospitals					
Primary hospitals and below *n* = 61 (10.83%)	1594.76 (949.61, 2174.67)	370.57 (275.46, 670.16)	333.45 (231.96, 598.03)	29.00 (15.95, 72.49)	192.49 (133.38, 317.50)
Secondary hospitals *n* = 188 (33.39%)	1994.89 (1406.29, 3979.64)	686.10 (478.43, 1232.31)	577.90 (388.94, 1137.66)	86.99 (57.99, 167.81)	345.20 (239.21, 594.68)
Tertiary hospitals *n* = 172 (30.55%)	1739.74 (1144.17, 2899.56)	1757.94 (988.65, 2796.03)	1377.68 (711.42, 2240.09)	289.96 (144.98, 507.42)	1126.48 (546.19, 1803.45)
Unknown *n* = 142 (25.22%)	1739.74 (1304.80, 2413.88)	283.00 (144.98, 563.61)	188.47 (101.48, 434.93)	44.94 (29.00, 144.98)	231.96 (137.73, 529.17)
*Z*	15.104	−11.759	−12.415	−5.417	−8.703
*p* value	0.000^*∗*^	0.000^*∗*^	0.000^*∗*^	0.000^*∗*^	0.000^*∗*^

^a^Low-economic region: Shufu, Wushi, and Atushi; Middle-economic region: Huocheng, Emin, and Yanqi; High-economic region: Yizhou District. ^*∗*^*p* value <0.05.

**Table 4:** OOP proportions among hospitals.

Hospitals	OOP/inpatient costs (%)	OOP/outpatient costs (%)	OOP/medical costs (%)
Primary hospitals and below *n* = 61 (10.83%)	20.23 (17.66, 30.25)	100.00 (100.00, 100.00)	51.28 (26.95, 61.48)
Secondary hospitals *n* = 188 (33.39%)	31.53 (20.13, 41.42)	100.00 (100.00, 100.00)	39.97 (28.59, 66.60)
Tertiary hospitals *n* = 172 (30.55%)	45.82 (25.67, 59.14)	100.00 (100.00, 100.00)	52.38 (35.41, 65.45)
Unknown *n* = 142 (25.22%)	93.34 (80.60, 100.00)	100.00 (76.70, 100.00)	100.00 (78.46, 100.00)
*Z*	56.473	3.218	−4.630
*p*	0.000^*∗*^	0.359	0.000^*∗*^

^*∗*^ *p* value <0.05.

## Data Availability

The datasets generated and/or analyzed during the current study are available upon reasonable request from the corresponding author.

## Abbreviations

OOP: Out-of-pocket
IQR: Interquartile ranges
NNDRS: National notifiable disease reporting system
USD: United States dollar
CNY: Chinese yuan
SES: Socioeconomic status.

## Supplementary Materials

Supplementary Materials Supplementary Appendix 1. Utilizing latent class analysis (LCA) for evaluating socioeconomic status (SES) of patients (Supplementary File 1). STROBE Statement-Checklist of items that should be included in reports of cross-sectional studies (Supplementary File 2).

## References

[1] Franco M. P., Mulder M., Gilman R. H., Smits H. L. (2007). Human brucellosis. *The Lancet Infectious Diseases*.

[2] Pappas G., Akritidis N., Bosilkovski M., Tsianos E. (2005). Brucellosis. *New England Journal of Medicine*.

[3] Pappas G., Papadimitriou P., Akritidis N., Christou L., Tsianos E. V. (2006). The new global map of human brucellosis. *The Lancet Infectious Diseases*.

[4] Yang H., Chen Q., Li Yu, Mu Di, Zhang Y., Yin W. (2023). Epidemic characteristics, high-risk areas and space-time clusters of human brucellosis-China, 2020-2021. *China Cdc Weekly*.

[5] Al Jindan R. (2021). Scenario of pathogenesis and socioeconomic burden of human brucellosis in Saudi Arabia. *Saudi Journal of Biological Sciences*.

[6] Dean A. S., Crump L., Greter H., Hattendorf J., Schelling E., Zinsstag J. (2012). Clinical manifestations of human brucellosis: a systematic review and meta-analysis. *PLoS Neglected Tropical Diseases*.

[7] Yang Y., Man X., Yu Z. (2022). Managing urban stroke health expenditures in China: role of payment method and hospital level. *International Journal of Health Policy and Management*.

[8] Li S., Chen Q., Yin W., Li Yu, Mu Di, Li Z. (2019). Diagnosis performance of brucellosis in China,2013-2018. *Disease Surveillance*.

[9] Liu Y., Xu C.-H., Wang X.-Mo (2020). Out-of-pocket payments and economic consequences from tuberculosis care in eastern China: income inequality. *Infectious Diseases of Poverty*.

[10] Marmot M. (2001). Inequalities in health. *New England Journal of Medicine*.

[11] Singh B. B., Khatkar M. S., Aulakh R. S., Gill J. P. S., Dhand N. K. (2018). Estimation of the health and economic burden of human brucellosis in India. *Preventive Veterinary Medicine*.

[12] Charypkhan D., Sultanov A. A., Ivanov N. P., Baramova S. A., Taitubayev M. K., Torgerson P. R. (2019). Economic and health burden of brucellosis in Kazakhstan. *Zoonoses and Public Health*.

[13] Zhang C., Gao H., Lin S. (2022). Medical costs of brucellosis patients in Xinjiang Uygur autonomous region, 2017-2019. *Chinese Journal of Epidemiology*.

[14] Wenti Xu, Jie Lv, Liu Y., Cheng Su, Dong X., Lin Li (2018). Analysis of economic burden on brucellosis patients in tianjin city from 2012-2015. *Chinese Journal of Infectious Diseases*.

[15] Jinping Li, Xuehua Xu, Zhu J., Wenhai D., Qiyuan L. (2019). Analysis on the epidemiological characteristics and burden of brucellosis, Laizhou city, 2012-2015. *Preventive Medicine Tribune*.

[16] Liu Wu, Yang L., Zeng T., Li L., Gao G., Liu H. (2018). The burden of brucellosis in Jingyuan County of Gansu Province and influencing factors. *Chinese Journal of Endemiology*.

[17] National Health Commission of the People’s Republic of China (2019). *WS 269-2019 Diagnosis for Brucellosis*.

[18] Kagamimori S., Gaina A., Nasermoaddeli A. (2009). Socioeconomic status and health in the Japanese population. *Social Science & Medicine*.

[19] Sartipi M., Nedjat S., Mansournia M. A., Baigi V., Fotouhi A. (2016). Assets as a socioeconomic status index: categorical principal components analysis vs. Latent class analysis. *Archives of Iranian Medicine*.

[20] Li C. (2005). Prestige stratification in the contemporary China: occupational prestige measures and socio-economic index. *Sociological Research*.

[21] Statistic Bureau of Xinjiang Uygur Autonomous Region (2023). *Xinjiang Statistical Yearbook 2021*.

[22] (2023). The People’s Bank of China. Exchange rate in 2020. https://www.boc.cn/sourcedb/whpj/.

[23] Lokamar P. N., Kutwah M. A., Atieli H., Gumo S., Ouma C. (2020). Socio-economic impacts of brucellosis on livestock production and reproduction performance in Koibatek and Marigat regions, Baringo County, Kenya. *BMC Veterinary Research*.

[24] National Health Commission of the People’s Republic of China (2021). *2021 China Health Statistics Yearbook*.

[25] Medical Security Bureau of Xinjiang Uygui Autonomous Region (2023). Circular on the Issuance of the Fourteenth Five-Year Plan for Medical Security in the Xinjiang Uygur Autonomous Region. Xinjiang: The People’s Government of Xinjiang Uygur Autonomous Region. https://ylbzj.xinjiang.gov.cn/ylbzj/ybfzgh/202201/f601bdf44c664e40947460fdc02ef04e.shtml.

[26] Medical Security Bureau of Xinjiang Uygui Autonomous Region (2023). Notice on the standardization of the outpatient slow and special disease coverage system for basic medical insurance in the region. https://ylbzj.xinjiang.gov.cn/ylbzj/dyb/202312/3325ad7504274309b934443b45988fbc.shtml.

[27] National Bureau of Statistics of China (2020). *China Statistical Yearbook 2020*.

[28] McDermott J., Grace D., Zinsstag J. (2013). Economics of brucellosis impact and control in low-income countries. *Revue Scientifique et Technique de l’OIE*.

[29] Zhang Y., Zhang C., Wang Z. Q. (2019). Research on the equity and influencing factors of health resource allocation in Xinjiang from 2004 to 2016: a comprehensive perspective based on population fairness and geographical equality. *Chin. Health Serv. Manag*.

[30] Huo X., Chen Li-L., Hong L. (2016). Economic burden and its associated factors of hospitalized patients infected with A (H7N9) virus: a retrospective study in Eastern China, 2013-2014. *Infectious Diseases of Poverty*.

[31] National Healthcare Security Administration (2023). Statistical bulletin on the development of the national health security service in 2020. https://www.nhsa.gov.cn/art/2021/6/8/art_75232.html.

