# Supplementary material for: The Economic Burden of Brucellosis Care in China: Socioeconomic Status Inequality
Source: J Trop Med. 2024 Aug 3;2024:7992287. doi: 10.1155/2024/7992287 (PMC11316907; doi:10.1155/2024/7992287)
Supplement: Supplementary Materials — Supplementary Appendix 1. Utilizing latent class analysis (LCA) for evaluating socioeconomic status (SES) of patients (Supplementary File 1). STROBE Statement-Checklist of items that should be included in reports of cross-sectional studies (Supplementary File 2). [file 7992287.f1.zip › Supplementary File 1.docx]

**Supplementary Appendix 1. Utilizing latent class analysis (LCA) for evaluating socioeconomic status (SES) of patients.**

The SES of individuals, which encompasses their access to material, human, and social capital, has a significant impact on their health outcomes. Education, employment, and income are recognized as crucial indicators of SES, and by taking these factors into account, we can create a comprehensive index to measure SES [1]. In this study, Latent Class Analysis was employed to construct the SES index.

Latent Class Analysis (LCA) is a statistical method used to uncover latent, unobserved classes within a population, based on a set of observed indicators or variables. It employs pattern recognition techniques to infer the underlying class structure. The process of Latent Class Analysis involves three steps. First, it requires the identification of observable or external variables. These variables serve as indicators for the latent classes being sought. Second, the modeling stage begins by assuming the presence of only one category and then progressively adding additional categories. Model comparisons are performed using statistical criteria to determine the optimal number of categories. Third, once the optimal number of categories is determined, the identified classes are named or labeled for further interpretation and analysis.

The study was conducted in three steps:

1. Identify the observables and external variables.

Based on an analysis of China's specific national circumstances and insights from pertinent literature, we have established a system for categorizing occupations and education levels, which effectively captures their respective degrees of prestige. In addition, we utilized the 4-quartile approach to categorize income levels. The resulting classification has been presented in Table 1 [2].

2. Modeling

In order to accommodate the discrete nature of the variables, a Latent Class Analysis (LCA) was conducted on data collected from 563 participants using RX64 4.3.0 Software [3]. The LCA modeling process began with a single category and progressively increased the number of categories. Model comparisons were then performed to determine the optimal number of categories. The model that yielded the lowest values for the Akaike Information Criterion (AIC) and Bayesian Information Criterion (BIC) was selected as the preferred model, as a lower AIC or BIC indicates a better model fit. Table 2 displays the results, where the model with 3 classes demonstrated the lowest BIC (3117.347), coupled with a relatively low AIC. Consequently, the model with 3 classes was chosen for further analysis (see Fig1).

Table 1. Classification by occupation, culture, and income level.

| level | Variables | | |
| --- | --- | --- | --- |
|  | Occupations | Education levels | Income |
| 1 | Jobless | Primary school and below | Q1 |
| 2 | Farmer and Herdsman | Junior high school | Q2 |
| 3 | Livestock-related workers and Veterinarian | High school/  Technical secondary school | Q3 |
| 4 | Civil servants, teachers, doctors, etc. | College degree or above | Q4 |

A higher rank indicates a more favorable social and economic situation.

Abbreviations: Q1 1st Quartiles, Q2 2nd Quartiles, Q3 3rd Quartiles, Q4 4th Quartiles

Table 2. The model evaluation results of latent class analysis.

| **Indicator** | **Classification** | | | |
| --- | --- | --- | --- | --- |
|  | **1** | **2** | **3** | **4** |
| **AIC** | 3342.937 | 3197.004 | 3117.347 | 3119.832 |
| **BIC** | 3381.937 | 3279.337 | 3243.012 | 3288.830 |
| **Proportions** |  | 0.8348/0.1652 | 0.2540/0.6057/0.1403 | 0.2540/0.0142/0.5915//0.1403 |

BIC Bayesian information criterion, AIC Akaike information criterion


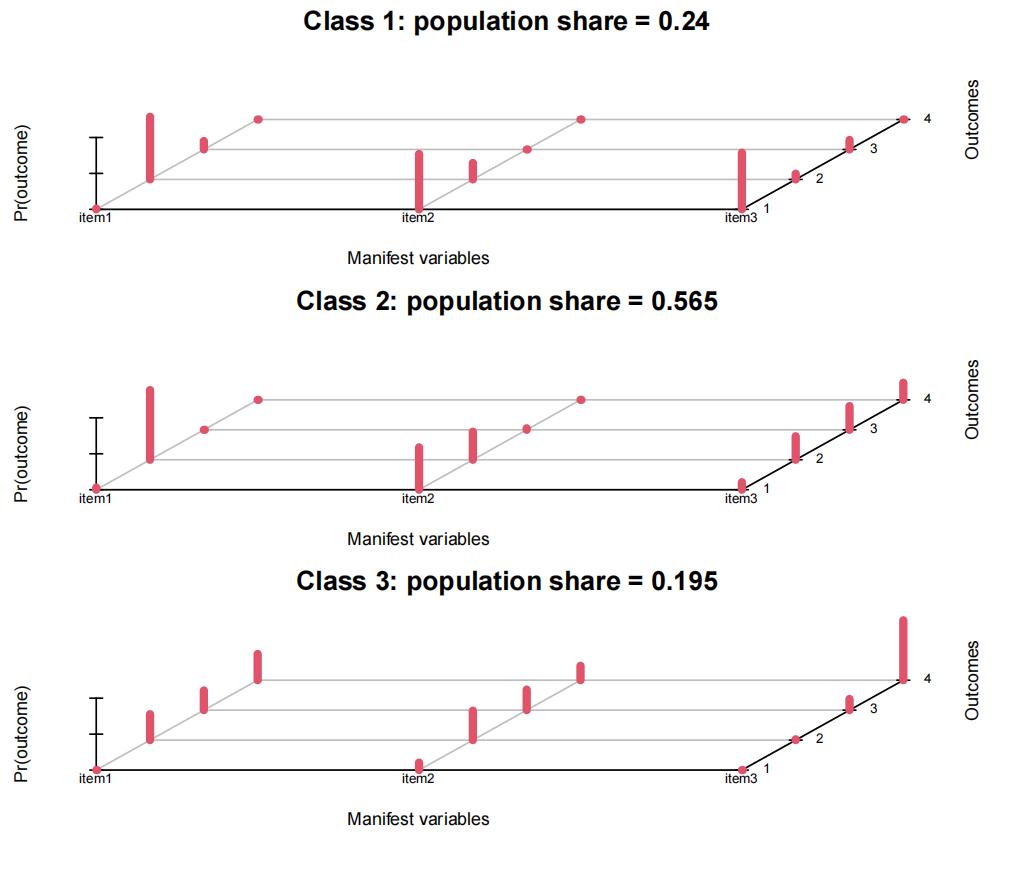


Fig. 1 The plot of the probability distribution of three latent variables

Item 1 Occupations; Item 2 Schooling; Item 3 Per capita annual household Income

3. Categorization:

Upon further examination of the probability distribution plot, distinct socio-economic classes can be identified. Class 1 comprises primarily of farmers and herders, who tend to have lower education levels and earn most of their income from the lowest tier. As a result, this class is categorized as "low SES" (socioeconomic status) due to its socio-economic profile. In contrast, Class 2, also consisting predominantly of farmers and herders, demonstrates higher levels of literacy and income. Thus, this class is appropriately referred to as "medium SES" to denote its intermediate socio-economic characteristics. Lastly, individuals in Class 3 are distinguished by their occupational status and literacy, with income concentrated at the highest levels. This particular class is labeled as "high SES" owing to its elevated socio-economic standing.

**References**

1. Kagamimori, Sadanobu et al. “Socioeconomic status and health in the Japanese population.” Social science & medicine (1982) vol. 68,12 (2009): 2152-60. doi:10.1016/j.socscimed.2009.03.030
2. Li, Chunling. “Prestige Stratification in Contemporary China: Occupational prestige measures and socioeconomic index.” Sociological Study vol. 02 (2005): 74-102+244. doi:10.19934/j.cnki.shxyj.2005.02.004.
3. Sartipi, Majid, et al. “Assets as a Socioeconomic Status Index: Categorical Principal Components Analysis vs. Latent Class Analysis.” Archives of Iranian medicine vol. 19,11 (2016): 791-796.
