# Supplementary material for: The Economic Burden of Brucellosis Care in China: Socioeconomic Status Inequality
Source: J Trop Med. 2024 Aug 3;2024:7992287. doi: 10.1155/2024/7992287 (PMC11316907; doi:10.1155/2024/7992287)
Supplement: Supplementary Materials — Supplementary Appendix 1. Utilizing latent class analysis (LCA) for evaluating socioeconomic status (SES) of patients (Supplementary File 1). STROBE Statement-Checklist of items that should be included in reports of cross-sectional studies (Supplementary File 2). [file 7992287.f1.zip › Supplementary File 2.docx]

**STROBE Statement-Checklist of items that should be included in reports of cross-sectional studies**

| **Item** | **Item No** | **Recommendation** | **Page Number** |
| --- | --- | --- | --- |
| **Title and abstract** | 1 | (a) Indicate the study’s design with a commonly used term in the title or the abstract | 1-2 |
|  |  | (b) Provide in the abstract an informative and balanced summary of what was done  and what was found | 1-2 |
| **Introduction** |  |  |  |
| Background/rationale | 2 | Explain the scientific background and rationale for the investigation being reported | 2-4 |
| Objectives | 3 | State specific objectives, including any prespecified hypotheses | 4 |
| **Methods** |  |  |  |
| Study design | 4 | Present key elements of study design early in the paper | 4-5 |
| Setting | 5 | Describe the setting, locations, and relevant dates, including periods of recruitment,  exposure, follow-up, and data collection | 4-6 |
| Participants | 6 | (a) Give the eligibility criteria, and the sources and methods of selection of  participants | 6-7 |
| Variables | 7 | Clearly define all outcomes, exposures, predictors, potential confounders, and effect  modifiers. Give diagnostic criteria, if applicable | 7-8 |
| Data sources/  measurement | 8 | For each variable of interest, give sources of data and details of methods of assessment (measurement). Describe comparability of assessment methods if there is more than one group | 7-8 |
| Bias | 9 | Describe any efforts to address potential sources of bias | 7-8 |
| Study size | 10 | Explain how the study size was arrived at | 6 |
| Quantitative variables | 11 | Explain how quantitative variables were handled in the analyses. If applicable, describe which groupings were chosen and why | 9 |
| Statistical methods | 12 | (a) Describe all statistical methods, including those used to control for confounding | 9 |
|  |  | (b) Describe any methods used to examine subgroups and interactions | 9 |
|  |  | (c) Explain how missing data were addressed | Not applicable |
|  |  | (d) If applicable, describe analytical methods taking account of sampling strategy | Not applicable |
|  |  | (e) Describe any sensitivity analyses | Not applicable |
| **Results** |  |  |  |
| Participants | 13 | (a) Report numbers of individuals at each stage of study—eg numbers potentially eligible, examined for eligibility, confirmed eligible, included in the study, completing follow-up, and analysed | 9-10 |
|  |  | (b) Give reasons for non-participation at each stage | 9-10 |
|  |  | (c) Consider use of a flow diagram | 10 |
| Descriptive data | 14 | (a) Give characteristics of study participants (eg demographic, clinical, social) and information on exposures and potential confounders | 9-13 |
|  |  | (b) Indicate number of participants with missing data for each variable of interest | Not applicable |
| Outcome data | 15 | Report numbers of outcome events or summary measures | 10-17 |
| Main results | 16 | (a) Give unadjusted estimates and, if applicable, confounder-adjusted estimates and their precision (eg, 95% confidence interval). Make clear which confounders were adjusted for and why they were included | Not applicable |
|  |  | (b) Report category boundaries when continuous variables were categorized | 11 |
|  |  | (c) If relevant, consider translating estimates of relative risk into absolute risk for a  meaningful time period | Not applicable |
| Other analyses | 17 | Report other analyses done—eg analyses of subgroups and interactions, and sensitivity analyses | Not applicable |
| **Discussion** |  |  |  |
| Key results | 18 | Summarise key results with reference to study objectives | 18 |
| Limitations | 19 | Discuss limitations of the study, taking into account sources of potential bias or imprecision. Discuss both direction and magnitude of any potential bias | 22-23 |
| Interpretation | 20 | Give a cautious overall interpretation of results considering objectives, limitations, multiplicity of analyses, results from similar studies, and other relevant evidence | 18-23 |
| Generalisability | 21 | Discuss the generalisability (external validity) of the study results | 18-23 |
| **Other information** |  |  |  |
| Funding | 22 | Give the source of funding and the role of the funders for the present study and, if applicable, for the original study on which the present article is based | 24 |
